# A Landscape Analysis of Supportive Supervisory Mechanisms in Maternal and Child Health Programs in India

**DOI:** 10.7759/cureus.86648

**Published:** 2025-06-24

**Authors:** Suruchi Gupta, Anirban Chatterjee, Kritika Singhal, Abhijit P Pakhare, Ankur Joshi

**Affiliations:** 1 International Health, Johns Hopkins Bloomberg School of Public Health, Johns Hopkins University, Baltimore, USA; 2 Community and Family Medicine, All India Institute of Medical Sciences, Bhopal, IND; 3 Health Services Policy and Management, University of South Carolina Arnold School of Public Health, Columbia, USA

**Keywords:** document analysis, health systems strengthening, lmics, maternal and child health, quality of care, supportive supervision

## Abstract

Supportive supervision of community health workers (CHWs) is considered a promising intervention for optimizing efficiency and health systems outcomes. However, little is known about how supportive supervision is conceptualized and implemented across maternal and child health (MCH) programs in India. We examined the scope of supportive supervision in selected MCH programs. We analyzed 29 programmatic documents and implementation guidelines from seven programs, and identified and extracted data on several themes, such as conceptualization, operationalization, monitoring and evaluation, and quality assurance of supportive supervision according to a pre-determined domain-based template. We found notable differences in the scope and quality of supportive supervision offered under different programs. Many programs did not include supportive supervision as a supervisory modality. Programs which included supportive supervision did not define all the parameters of operationalization - responsible workforce, frequency of visits, procedure, training, logistic support, supervision formats/checklists, scope for and frequency of monitoring and evaluation, and quality assurance of supervisory visits. Even when some parameters were defined, there was considerable heterogeneity in implementation across programs. Such inconsistencies may adversely affect the quality and efficiency of supportive supervision, thereby minimizing its potential. Policymakers should prioritize the redesign of supportive supervision strategies to ensure greater clarity and consistency across key domains, thereby optimizing community health worker performance and strengthening public health program outcomes.

## Introduction and background

Health systems in low and middle-income countries (LMICs) depend on community health workers (CHWs) to ensure last-mile coverage for clients. CHWs are indispensable to the public health system and are a crucial interface between the community and the healthcare system [1]. Therefore, sustained implementation of many public health programs impinges on their performance [2]. Ensuring uniform quality of care delivered through CHWs necessitates effective and sustained training, monitoring, and supervision. Although many factors influence the performance of CHWs, supportive supervision as a modality of effectively augmenting the performance of CHWs has gained traction in recent years [3-8]. As opposed to traditional models of top-down and authoritative methods of monitoring performance, supportive supervision encompasses a collaborative, participatory approach to problem-solving and skill-building [9]. Supportive supervision for CHWs has been associated with sustained and improved skillsets, increased interactions with beneficiaries, improved quality of work, and improved morale [10-13].

The Indian health system employs a range of CHWs, including Accredited Social Health Activists (ASHAs), Anganwadi Workers (AWWs), Multi-Purpose Workers (MPWs), and Peer Educators (PE/Saathiyas). With around 900,000 ASHAs and more than 130,000 AWWs, India has one of the largest CHW workforces in the world [14,15]. In recent years, supportive supervision has gained traction across maternal and child health (MCH) programs. For instance, a longitudinal multi-level analysis in Madhya Pradesh and Bihar found that higher-intensity supportive supervision was associated with a 70% increase in the odds of better CHW performance, both directly and via improved CHW knowledge [16]. Additionally, a mixed-methods field study from central India demonstrated that CHWs supervised by trained facilitators (“ASHA Sahyoginis”) scored significantly higher in knowledge and skills, affirming the link between supervisory support and CHW competency [17]. Pilot initiatives of digital supportive supervision tools in Rajasthan further suggest potential for scalable improvements in monitoring and service quality [18]. Supportive supervision is thus emerging as a critical and evidence-based strategy for enhancing CHW effectiveness in India’s MCH programs.

However, experience from other settings shows that the operationalization of supportive supervision varies significantly. Some countries have espoused a paradigm shift in implementing supervision to veer towards a more supportive and collaborative approach [19]. On the other hand, some countries see supervision as a method to implement regular, almost punitive visits to improve performance and ensure that CHWs do the assigned work [7]. In India, studies conducted in Odisha, Madhya Pradesh, and Bihar have mostly examined the impact of supportive supervision on AWWs, showing that supportive supervision improves CHW performance by improving CHW knowledge [16,20].

Few studies have examined the operationalization, governance structures, policy mandates, and approaches to implementing supportive supervision for ASHA-led MCH programs in India. Several systemic issues, including the lack of capacity of supervisors and the lack of logistic and institutional support, often hamper the operationalization of supportive supervision [7]. A thorough understanding of how supportive supervision is envisioned in program documents allows for the identification of implementation bottlenecks. Addressing these challenges is critical to accelerating progress toward Sustainable Development Goals (SDGs 3) targets, particularly SDG 3.1 (reducing the global maternal mortality ratio) and SDG 3.2 (ending preventable deaths of newborns and children under five years), by ensuring that frontline health workers are adequately supported to deliver high-quality MCH services. To understand these mechanisms and identify potential opportunities for strengthening the implementation of supportive supervision of ASHAs in national MCH programs, this study explores the research question: "How is supportive supervision conceptualized and operationalized across different MCH programs in India?"

This article was previously published as a preprint on the Preprints.org server on November 24, 2021, https://www.preprints.org/manuscript/202111.0454/v1.

## Review

Methodology

We conducted a document analysis to enumerate and illustrate supervisory methods embedded in existing guidelines for first-line supervisors in MCH programs.

Selection of Programs

We shortlisted health programs implemented by the Ministry of Health and Family Welfare (MoHFW) that were scaled across the country, provided client-facing health services, and prescribed ASHAs as the key cadre for service delivery. We selected a total of seven programs that target each demographic group in the MCH care continuum. Subsequently, we shortlisted two programs targeting pregnant women (Janani Shishu Suraksha Karyakram {JSSK}, Janani Suraksha Yojana {JSY}), two targeting infants (Home Based Neonatal Care {HBNC}, Home Based Care for Young Child {HBYC}), one targeting under five years (Universal Immunization Program {UIP}), one targeting children and adolescents (Rashtriya Bal Swasthya Karyakram {RBSK}), and one targeting only adolescents (Rashtriya Kishor Swasthya Yojana {RKSK}) [21-28]. These programs were chosen to represent each stage of the MCH continuum under NHM, and no other NHM programs met our inclusion criteria.

While the Integrated Child Development Services (ICDS) program plays a significant role in MCH through the work of AWWs, this paper focuses specifically on programs under the purview of the MoHFW. This focus was chosen to enable a more in-depth analysis of supervisory frameworks governing ASHAs, who are the primary CHW cadre supported directly by MoHFW initiatives. By narrowing the scope, we aimed to provide clearer insights into the design and implementation of supportive supervision mechanisms within health-sector-led MCH programs, while recognizing that cross-sectoral coordination with ICDS remains an important area for future inquiry.

Selection of Documents

We identified 29 documents for the review within the selected programs. These were implementation documents, such as guidelines, operational manuals, and/or training modules. These documents were extracted from various online public repositories, such as those of the National Health Mission, Government of India (NMH, GoI), the MoHFW (GoI), and dedicated programmatic websites (when available).

Synthesizing Domains and Framework for Extraction of Relevant Information

We retrieved existing literature and enlisted potential domains affecting the quality of supervision based on the WHO Guidelines for training of mid-level managers in supportive supervision [7,9,29]. For the purpose of this study, supportive supervision was defined as follows: a facilitative process that promotes problem-solving, two-way communication, mentoring, and capacity-building between supervisors and frontline workers, with the goal of improving service delivery and worker performance.

A panel of four subject matter experts, each with over a decade of experience designing, implementing, or evaluating community health programs involving CHWs, was convened to review the literature and guidelines. Through structured discussions, the panel iteratively refined the domain framework and finalized six domains considered most critical for assessing supportive supervision in the Indian context. These were as follows: programmatic commitment, workforce, operational details, incentivization, monitoring and evaluation, and quality assurance.

To reduce bias, two independent reviewers (authors SG and AC) conducted an initial round of document analysis using a standardized extraction template based on the six domains. A third author (AJ) reviewed the extracted data for completeness and consistency. Disagreements were resolved through discussion until consensus was achieved. This triangulated approach enhanced reliability and mitigated individual bias in interpretation.

Since the article was published on the preprint server, the authors have substantially revised the analytical framework. Specifically, the domain synthesis was restructured to improve conceptual clarity and policy relevance, for example, by combining overlapping constructs, integrating emerging evidence, and aligning domain definitions more closely with WHO and MoHFW priorities. While the programs and documents analyzed remained the same, the renewed analysis involved recoding data according to the refined framework, enabling a more nuanced yet clearer interpretation of supervisory mechanisms. These revisions have improved the applicability of the findings to inform ongoing program design and policy discussions.

Results

We reviewed 29 documents from seven national health programs for this landscape analysis (Table 1). For each program, operational specifications of supportive supervision were described under six identified domains (Table 2).

**Table 1:** A summary of the documents reviewed for landscape analysis of supportive supervision in maternal and child health programs in India. *Program targets both children under five years and school-going adolescents. ASHA: Accredited Social Health Activist; RMNCH: Reproductive, Maternal, Newborn, and Child Health; PIB: Press Information Bureau; DPM: Press Information Bureau; MoHFW: Ministry of Health and Family Welfare; NHM: National Health Mission; NRHM: National Rural Health Mission; ANM: Auxiliary Nurse Midwife; LHV: Lady Health Visitor

Target beneficiary	Name of the health program	Total documents reviewed	Guidelines/operational guidelines	Handbooks/training handbooks	Modules/training modules	Other documents	Name of the documents
Pregnant women	Janani Shishu Suraksha Karyakram (JSSK)	7	1	0	1	5	Guidelines for JSSK (June 2011), JSSK dietary norms (July 2018), induction training module for ASHAs, Maternal and Newborn Health Toolkit (January 2013), RMNCH+A Supportive Supervision Plan and Checklists (2013), Janani Shishu Suraksha Karyakram - press release (PIB - March 13, 2020), job responsibilities - DPM
	Janani Suraksha Yojana (JSY)	2	1	0	0	1	Janani Suraksha Yojana: guidelines for implementation, Janani Suraksha Yojana: features and frequently asked questions
Newborns and infants	Home Based Newborn Care (HBNC)	2	1	1	0	0	Home Based Newborn Care Operational Guidelines (revised 2014), Handbook for ASHA Facilitators
	Home Based Care for Young Child (HBYC)	2	1	1	0	0	Home Based Care for Young Child (HBYC) - strengthening of health and nutrition through home visits: operational guidelines (April 2018), Handbook for ASHA on Home Based Care for Young Child: additional home visits to address the young child
Under five years	Universal Immunization Program (UIP)	2	0	2	0	0	Immunization Handbook for Health Workers - 2018, Immunization Handbook for Medical Officers (reprint 2017), Universal Immunization Program - MoHFW
	Rashtriya Bal Swasthya Karyakram (RBSK)*	6	3	1	0	2	Background and rationale - RBSK (NHM), Rashtriya Bal Swasthya Karyakram (RBSK) - child health screening and early intervention services under NRHM: Operational Guidelines (October 2013), setting up district early intervention centres: Operational Guidelines (May 2014), guiding note for Early Childhood Development (ECD) call centre: Operational Guideline (2019), RBSK Participant’s Manual for Mobile Health Teams (July 2014), Helping ASHAs identify birth defects
Adolescents	Rastriya Kishore Swasthya Karyakram (RKSK)	8	2	1	3	2	Guidelines for implementation of RKSK (March 2015), RKSK Operational Framework: translating strategy into programs (January 2014), Adolescent Health and Wellness Days “Yuva Samvad”, RKSK Strategy Handbook (January 2014), Implementation Guidelines (RKSK) (2018), RKSK Facilitator’s Guide: training module for ANMs/LHVs, RKSK Facilitator’s Guide: training module for Medical Officers, RKSK Facilitator’s Guide: training module for Peer Educators

**Table 2:** Domains and categories under which supportive supervision has been described. CHWs: community health workers; ASHAs: Accredited Social Health Activists; AWWs: Anganwadi Workers; PEs: Peer Educators; ANM: Auxiliary Nurse Midwife

Domains	Categories
Programmatic commitment	Mandate for supportive supervision: availability of documents on operationalization of supportive supervision in the health program at the community level, with or without specific documents or sections dedicated to supportive supervision.
Workforce	Identified primary supervisor cadre: cadre of healthcare workers responsible for direct supervision of CHWs (ASHAs/AWWs/PEs/ANMs).
	Identified additional supervisor cadre: a cadre of healthcare workers who would be responsible for assisting the primary supervisor cadre in undertaking supportive supervision measures according to the health program guidelines. This supportive cadre may themselves hold supervisory roles over the primary cadre, but their function in this context is primarily to provide support and troubleshoot when needed.
	Capacity building: provision of training for primary and secondary cadre in supportive supervision.
Operational details	Frequency: refers to the periodicity of supportive supervision (‘when’) as specified by the programmatic documents.
	Key objectives of supervision: refer to the program-specific objectives (‘why’) of supportive supervision.
	Activities to be supervised: refers to the specific activities (‘what’) by CHWs which warrant supportive supervision according to the programmatic documents.
	Means of conducting supportive supervision: refers to process guidelines, checklists, mobile phone applications, and/or other similar modalities (‘how’) for standardizing supportive supervision.
	Systematic or comprehensive supportive supervision: provision for combining supportive supervision with other concurrently ongoing activity(s).
Incentivization	Monetary incentives for supportive supervision: includes travel allowances, mobility support, and performance-based incentivization for the primary supervision cadre.
	Non-monetary incentives for supportive supervision: includes social recognition, accolades, training, opportunities for growth and advancement.
Monitoring and evaluation	Framework for reporting and monitoring supervision: clear guidelines on the process to be followed to monitor implementational fidelity of supportive supervision.
	Measurement of supervisory activity: Health Management and Information System (HMIS) indicator(s) for recording activities undertaken as a part of supportive supervision, including but not limited to attendance, quality, competency assessment, etc.
Quality assurance	Compliance with supportive supervision standards: whether programmatic understanding meets the principles of supportive supervision, i.e., it is focused on building relationships and improving performance, provides respectful constructive feedback, espouses a mentor-mentee relationship, uses local evidence to drive positive change, and involves regular follow-up.
	Dashboard to assess gaps: provision of a dashboard to reflect supportive supervision metrics, including but not limited to the number of supervisory visits conducted, number of supervisory visits completed, number of reports submitted, CHW satisfaction, etc.

Programmatic Commitment

Fifteen programmatic documents on HBYC (1), HBNC (1), JSSK (5), RBSK (2), UIP (2), and RKSK (4) specifically mentioned “supportive” supervision, while JSY only mentioned supervision. Three of these - Immunization Handbook for Medical Officers (UIP), Handbook for ASHA facilitators (HBNC), and Operational Framework (RKSK) - have sections outlining the scope and key activities of supportive supervision.

Workforce

Identification of the primary supervisory cadre varied considerably across programs. In the HBNC and HBYC programs, ASHA facilitators supervised their peers, while in the RBSK and JSSK programs, the supervisors were higher-level officials such as the Block Medical Officer (BMO) and the District Program Manager (DPM), respectively. One program (RKSK) did not specify any primary supervisory cadre. It instead earmarked two CHWs - ASHAs to act as the first-level resource persons and ANMs to monitor and train the Peer Educators monthly. Prescribing additional supervisory cadres, which can allow for task-sharing, is also not mentioned in any of the programmatic documents. Only two programs, HBYC and RKSK, stipulated training for supportive supervision. HBYC mandates a supervisory period of two days for enhancing supervisory skills. RKSK does not specify the duration of training; however, it mandates training of ASHAs as PE coordinators.

Operational Details

Mandated frequency of supervisory visits ranged from twice weekly (for DPMs in JSSK) to at least once every quarter (HBYC). The frequency of supportive supervision was not mentioned in the reviewed programmatic documents of RBSK. Some programmatic documents, such as the Handbook for ASHA Facilitators (HBNC) and the Immunization Handbook for Medical Officers (UIP), enlisted mentoring, support, and problem-solving as the key objectives of undertaking supportive supervision. However, documents for JSSK and JSY did not identify any such key objectives. Although most programmatic documents identified activities that needed supportive supervision, those for JSSK did not. Several programmatic documents provided checklists to facilitate the process of supportive supervision in addition to regular meetings. Two programs (RBSK and JSSK) did not provide any tools for enabling supportive supervision. The other programs provided one or more of a variety of tools, including SOPs, monitoring formats, checklists, job aids, and training materials, to support and standardise supportive supervision. Most programmatic documents directed supportive supervisory visits to be carried out during the Village Health, Sanitation and Nutrition Days (VHSNDs), whereas documents for JSSK and JSY did not. For UIP, supportive supervision is mandated during routine immunization sessions or Supplementary immunization activities (Mission Indradhanush).

Incentivization

Only two programs provided monetary incentives for the primary supervisory cadre: HBYC and RKSK. HBYC fixed a flat incentive amount of Rs. 500 per month for the primary supervisory cadre (ASHA facilitator/ANM/Anganwadi services supervisor). No fixed incentive amount was stipulated in RKSK. Instead, the district-level officials were given the flexibility to determine the incentive amount. No travel allowances or mobility support were stipulated for any primary supervisory cadre. However, UIP generally provides mobility support for district and state-level officials. None of the programs offered non-financial incentives.

Monitoring and Evaluation

Only one program (HBYC) had a specific HMIS indicator for supportive supervision, i.e., the number of ASHAs who received supervisory visits. No other program explicitly described monitoring and evaluation procedures to measure supportive supervision frequency, processes, or outcomes. No program prescribes the use of or advocates for a data dashboard to enable continuous monitoring.

Quality Assurance

The WHO module for supportive supervision for mid-level managers has set parameters and characteristics of supportive supervision [9]. HBNC and UIP guidelines meet these parameters and outline the desired characteristics of a supportive supervisor. UIP also provides a supervisory matrix to facilitate a step-by-step approach to supportive supervision. No program explicitly described quality assurance procedures for supervision.

Among the reviewed programs, HBNC, HBYC, and UIP had the most comprehensive supportive supervision strategies that covered multiple domains. They defined supervisory cadres, visit frequency, and quality assurance clearly, while aligning closely with WHO best practices. In contrast, programs like JSSK, JSY, and RBSK provided minimal or no specific guidance on supportive supervision, lacking clarity on operational roles or monitoring mechanisms.

Discussion

Supportive supervision is an effective strategy to motivate CHWs, improve their performance, and consequently lead to a higher CHW retention rate [30,31]. It is also recommended as a mode of supervision in several programmatic documents that we reviewed. However, in our review, we found notable inconsistencies in its conceptualization that affected its operationalization in MCH programs.

Most programmatic documents did not specify capacity-building measures for training supportive supervisors. Providing standardized training to supervisors and on-the-job support using digital health tools boosts the quality of supervision and improves the performance of supervisees [32-34]. On the other hand, inadequate technical support for training and capacity building often leads to poor understanding of supportive supervision, leading to differences in implementation and suboptimal outcomes [7,35,36].

None of the reviewed documents specified standardized operational procedures (SOPs) or job aides to assist supportive supervision. Only the programmatic document on the immunization program (UIP) provided specific job aides for on-spot troubleshooting. Importantly, there were no guidelines or SOPs that define mechanisms of providing feedback to supervisees - a core component of supportive supervision. Providing well-defined job roles, responsibilities, and SOPs of supportive supervision can augment its quality and overall productivity [31,33,37].

Evidence suggests that the primary cadre responsible for supportive supervision is often tasked with additional administrative activities, which can be time-consuming [19,38]. The remaining and limited supervisory time affects the quality and implementation of supportive supervision [19,39,40]. Therefore, there is a need to objectively and explicitly identify time management and related competencies as necessary prerequisites in the training manuals for supportive supervisors.

None of the programmatic documents specified any provision for logistic support to facilitate supportive supervision. Logistic constraints, such as lack of incentives and limited transportation, are common across healthcare settings in LMICs [8,33,37,38,41]. Supportive supervisors usually have to travel long distances for field visits and on-site supervision, and the lack of dependable transport can reduce the frequency of supervision [42-44]. Non-payment or delayed payment of incentives also impacts the morale of the supervisors [19,42,44]. Incentivization of supportive supervision and provision for transportation, especially for hard-to-reach areas, is needed to encourage regular and quality implementation. Additionally, identifying additional supervisory cadres who can assist with task-sharing can address constraints due to limited staff availability and transportation [7].

Due to resource limitations, most programmatic documents prescribe that supportive supervision be conducted alongside other routine health activities. In practice, this often results in a single supervisor being responsible for conducting multiple evaluations during VHSNDs, including immunization reviews, nutrition counseling, and check-ups, each requiring a separate checklist. Despite recording overlapping parameters, such as ASHA attendance or stock availability, these checklists are formatted differently across programs (e.g., UIP, HBYC, and JSSK), leading to duplication of effort and increased paperwork. While multitasking is often unavoidable in such settings, it significantly adds to the workload and dilutes the quality of supervision [41,45-48]. On the other hand, in resource-limited healthcare settings, multitasking is one of the few ways of ensuring any supervision at all. In such settings where supervisory cadres frequently operate under intense workload pressures, inadequate transportation, and limited digital infrastructure, even uniformity in guidelines alone cannot guarantee effective field-level implementation. Given this dichotomy, performance evaluation that focuses on a few key indicators would help reduce the workload of the supportive supervisors. Digital dashboards may enable self-monitoring by supervisors and improve efficiency.

Several factors, such as low coverage, lack of motivation, and poor staff attitude, limit the effectiveness of supportive supervision in LMICs [7,8,49-52]. Despite this, the documents reviewed did not emphasize the importance of these factors, highlighting the pressing need to include these components in SOPs to ensure high-quality supervision. Focusing on the quality of each supervisory visit would be more beneficial than increasing the total number of visits [8]. On the other hand, lengthy supportive supervisory sessions may prove counterproductive [8,53,54]. Therefore, a balance must be struck between the number of visits and the tasks performed at each visit. Quality supportive supervision would include recording a limited but meaningful number of indicators to quantify performance while providing avenues for overall collaborative learning and growth.

Many of these observed inconsistencies may be attributed to historical context, health systems structure, and program type. Programs, such as JSY and JSSK, were introduced in the mid-2000s, when supportive supervision had not yet gained prominence as a recommended approach. In contrast, more recent initiatives like the HBYC and the RKSK reflect a growing recognition of supportive supervision as a tool for performance enhancement and health system strengthening. In addition to these temporal differences, health governance and institutional fragmentation play a significant role. Many public health programs in India are implemented as vertical initiatives, often with separate planning, monitoring, and training systems. This can lead to variation in how supervision is operationalized. Without a standard framework, each program tends to adopt its own interpretation of supervision, which may or may not align with evidence-based best practices. India’s federated structure also allows states significant autonomy in the adaptation and implementation of national health programs. While national guidelines provide a uniform policy framework, implementation varies widely across states due to differences in state-level leadership, workforce availability, budgetary priorities, and institutional readiness. This decentralization, while enabling contextual adaptation, also leads to heterogeneity in supervision quality, with some states innovating beyond national guidelines and others having limited execution. Additionally, programs like HBYC and HBNC, which involve intensive community outreach and are often supported by development partners, tend to include more detailed supervision frameworks. In contrast, schemes like JSY, which emphasize financial entitlements rather than service delivery, may view supervision as less central.

There is an urgent need to improve CHW-led programs in India, given the ongoing demographic expansion, rapid urbanization, and an impending health workforce shortage. Supportive supervision can act as an efficient and sustainable tool for CHW capacity building and improving efficiency [55]. Lessons can be drawn from other decentralized LMICs, such as Brazil, where the Family Health Strategy (FHS) uses a dedicated supervisory role embedded in multidisciplinary teams, with clear accountability and structured mentorship processes. Studies from Brazil show that consistent supervision linked to local health units improved CHW performance and community trust [56].

This study has a few limitations. First, the analysis was limited to national-level programmatic documents available in the public domain; sub-national adaptations or informal implementation practices were not captured. Second, while the framework was rigorously applied, interpretation of textual content may carry some subjectivity despite efforts to minimize bias through multiple reviewers. Third, all documents included in this analysis were publicly available and current as of November 2021. Any changes made to programmatic documents after this time are not reflected in the study. This represents a temporal limitation of the analysis. Finally, the study focused only on MoHFW-led programs and did not include cross-sectoral programs like ICDS, which may offer additional insights into supportive supervision strategies. Despite these limitations, this review is the first to explore the relationship between program mandates and the operationalization of supportive supervision for CHW-led programs.

## Conclusions

Despite supportive supervision being an effective approach for improving the performance of CHWs, there are significant inconsistencies in how it is conceptualized and implemented across different Indian health programs. Many program documents either omit supportive supervision entirely or provide incomplete operational guidelines. These gaps in implementation guidance likely reduce the effectiveness of supportive supervision and limit its potential benefits for CHW performance and program outcomes. To optimize CHW performance through supportive supervision, programs need clear, standardized guidelines that address critical components as follows: supervisor selection and training, visit procedures and frequency, evaluation methods, and quality assurance frameworks. However, strengthening supervision is not only about improving guidelines; it also requires addressing systemic constraints, such as workforce shortages, multitasking without role clarity, and inadequate logistical support. These systemic issues must be addressed through a systems-level approach that combines policy reform, capacity building, financing, and institutional accountability.

To address supportive supervision-specific challenges, programs should consider the development of a digital dashboard that enables real-time monitoring and accountability. This dashboard could capture key process and outcome indicators, such as the proportion of CHWs receiving at least one supervisory visit per quarter, the percentage of trained supervisors, supervisor-to-CHW ratios, the proportion of completed supervision checklists, and CHW-reported satisfaction with supervision. In parallel, there is an urgent need for a standardized training module for supervisors that is both practical and systems-oriented. Core components should include foundational principles of supportive supervision, clarity on roles and time management, techniques for giving constructive feedback and mentoring, the use of digital monitoring tools, and strategies to address logistical barriers, particularly in underserved or remote areas. These recommendations align closely with the WHO’s five-step supportive supervision model, which emphasizes structured planning, observation, analysis, feedback, and follow-up. Enhancing the consistency, quality, and accountability of supervision will be essential to improving CHW capacity and advancing maternal and child health outcomes across India.
